# Speech Restoration & Mental Surveillance as Conflicting Ethical Concerns in Neural Speech‐Decoding Research

**DOI:** 10.1002/hast.70090

**Published:** 2026-10-28

**Authors:** Narayan Sankaran, Winston Chiong

**Keywords:** speech brain‐computer interfaces, speech decoding, mental privacy, communicative intent, neuroethics, bioethics, neuroscience and society

## Abstract

*The research program of speech decoding—“reading” words directly from brain activity—elicits an unusually wide range of public responses, reflecting a tension between the ethical goal of restoring speech for people with serious communication disabilities and the ethical goal of protecting mental privacy from neurotechnological surveillance. These competing moral perspectives are informed by intrinsic features of the research but also by the broader sociocultural context in which neuroscience is conducted and is communicated to an increasingly interested public. Whereas prior neuroethical literature has looked “downstream” from this research in proposing governance frameworks, in this paper we turn “upstream” to the neuroscience of language to consider how ethical tensions may be informed by the specific neural circuits from which speech is inferred. Our analysis identifies the* communicative intent *of neural signals as a key feature of some but not all speech‐decoding strategies, and we argue that whether a speech BCI decodes linguistic information with or without communicative intent should inform assessments of the ethical implications of the research. At the level of public discourse, we show how such distinctions can inform responsible science communication about future advances in this fast‐moving field*.

## Article

With increasing speed and accuracy, neuroscientists can “read” words and sentences directly from patterns of brain activity measured while human research participants are speaking, attempting to speak, or merely imagining the act of speaking. This feat—termed “speech decoding”—is being facilitated by rapid advances in the neural interfaces that measure patterns of activity in brain regions associated with speech and language and by machine learning, which statistically infers information from those patterns. For many research programs in this domain, the end goal is a usable communication device—a speech brain‐computer interface (speech BCI)—that would decode users’ intended messages and generate text or audio output in real time, bypassing the need for vocalization altogether.^1^

As a neuroscientific research program, speech decoding elicits an unusually wide range of public responses encompassing both praise and fear (or even hostility), depending on the applications being envisioned and the aspects of research emphasized in public reporting. On one hand, for people who are paralyzed and cannot speak, speech BCIs show promise as an innovative neuroprosthetic that would restore the ability to fluently communicate. On the other hand, much of the public and academic discourse regards speech‐decoding research with trepidation, believing that it might yield technologies capable of reading private information from the brain. This dual perception is reflected in disparate appraisals of research offered by news reports, with some highlighting the promise of restoring lost function^2^ but others expressing alarm about the imminent loss of mental privacy.^3^ In this work, we will refer to the two envisioned applications that elicit such polarized responses as “restoration” and “surveillance,” respectively.

Here, we seek to understand the contexts under which such disparate reactions to research emerge, and we advance a framework for weighing the competing moral priorities being expressed. First, we offer conjectures about the different lenses through which people might view research, and we consider how these lenses might influence one's moral priorities when evaluating whether the benefits of restoration outweigh the risks of surveillance. Second, looking upstream, to the science of speech decoding, we propose a functional differentiation between speech BCIs based on whether they decode linguistic information with versus without *communicative intent*. We argue that the ethics of downstream uses will require reorienting both scientific research and public reporting of science around this distinction.

## Competing Moral Priorities

As responses to speech‐decoding research, support for restoration and concerns about surveillance both stem from a desire to promote or protect human interests, yet they prioritize different moral concerns. Specifically, support for restoration recognizes the potential for speech BCIs to facilitate social inclusion for people with serious communication disabilities. Conversely, objections based in concerns about surveillance prioritize the protection of fundamental human rights—such as the right to privacy and freedom of thought—against the long‐term threats posed by emerging technologies. How might these inverted moral schemas arise as a function of the lens through which research is viewed?

First, we consider the view that research is a vehicle for achieving restoration. By decoding users’ intended messages directly from brain activity, speech BCIs could provide a fluent communication channel to those who are unable to speak due to dysfunction arising from brain‐stem stroke, neurodegenerative diseases such as amyotrophic lateral sclerosis (ALS), or neurodevelopmental disorders such as cerebral palsy. The pathophysiology of these conditions often results in speech‐motor disorders such as anarthria (loss of control over vocal muscles responsible for articulation) or apraxia (loss of the ability to plan, sequence, and coordinate those articulatory movements). These disorders often affect speech‐motor function while sparing the higher‐order brain circuits that underlie faculties such as conceptual reasoning and language. As a result, many people with speech‐motor disorders will remain capable of formulating complex ideas and crafting specific messages but will lack the ability to physically articulate them.

For people affected by such conditions, a device capable of restoring speech‐based communication could considerably improve quality of life. The capacity to convey complex ideas via speech is vital for our agency and autonomy, facilitating a broad range of human endeavors from pursuing professional or educational opportunities to seeking care to forming and maintaining social bonds. The degree to which these pursuits are facilitated depends not just on whether speaker and listener can be mutually understood but also on whether such communication can unfold fluently—that is, in real time and with efficiency, precision, and relative ease.

Although a range of existing assistive and augmentative communication (AAC) devices are used by many people with physical disabilities, the communication afforded by these devices is generally inflexible, effortful, and slow, usually relying on users’ residual voluntary movements (such as finger pointing or eye or head movements) or on partner‐assisted scanning approaches. This results in rates of communication output below 15 words per minute,^4^ substantially less than the 150‐to‐200‐words‐per‐minute rate at which fluent speech unfolds.^5^ Existing AAC devices may therefore permit the transmission of basic messages but are unsuited to the rich real‐time dialogue that characterizes many meaningful and complex human exchanges, such as those shared among romantic partners, family, and close friends.

By contrast, a speech BCI that decodes users’ intended speech directly from brain activity could provide efficient real‐time access to the full expressive capabilities of language. This promise is not merely speculative; it reflects actual advances in the field. In a recent clinical trial, a patient with severe vocal paralysis used a speech BCI to accurately generate sentences at an average rate of 78 words per minute.^6^ While the speech‐BCI system in this study used a modest, closed set of words, more recent efforts have seen systems with 125,000‐word vocabularies achieving near‐perfect accuracy while maintaining high output rates.^7^ As the rapid pace of current progress reflects technical and neuroscientific advances for which further improvements are relatively projectable, we anticipate that the fluency of decoded outputs will continue to increase in coming years. Given the aforementioned importance of fluency in promoting users’ agency, autonomy, and overall quality of life, speech BCIs represent more than just incremental progress over existing AAC devices along technical dimensions such as speed or accuracy. This technology is likely to open entirely new doors of social and economic opportunity for patients with vocal paralysis. Indeed, research has found that prospective BCI users strongly desire assistive technologies that would enable real‐time direct interactions,^8^ prioritizing qualities such as speed and ease of use in a device^9^—a point that rehabilitation professionals have also made.^10^

Given the importance of fluent speech for promoting quality of life, the limited ability of existing AAC technologies to restore fluent speech when lost, and the promise of speech BCIs in this regard, one can appreciate how research is viewed as not only permissible but also commendable. One can understand, that is, how the acute and near‐term benefits associated with restoration would supersede speculative concerns about surveillance that might arise when considering future applications.

Perhaps for these reasons, support for restoration seems widespread, despite the relatively small proportion of the overall population afflicted by severe speech‐motor disorders. Narratives in mainstream media that shape public opinion about speech decoding—such as front‐page coverage in the *New York Times* ^11^—adopt a journalistic lens that underscores the promise of restoration. These narratives often center on specific individuals living with speech‐motor disorders, highlighting the challenges they face and showcasing the embedded use of speech BCIs as innovative neuroprostheses. For the vast majority of the public who are otherwise isolated from the daily experience of such individuals, these media portrayals provide a rare window into the day‐to‐day realities of being unable to speak and may powerfully bolster favorable attitudes toward speech‐decoding research.

Similar support for restoration pervades the scientific community, where, judging by the rationale provided in published research articles, the overwhelming majority of scientists seeking to develop speech‐BCI systems explicitly conceive of their work as fulfilling a moral obligation to people unable to communicate.^12^ These moral priorities expressed by scientists in turn reflect those established at higher levels of scientific governance. For instance, in the United States, a substantial portion of speech‐BCI research over the past decade has been funded through the Brain Research through Advancing Innovative Neurotechnologies (BRAIN) Initiative—a partnership between federal and nonfederal organizations—whose specific aims encompass the development of neurotechnologies for clinical treatments, including BCIs for speech‐motor disorders.^13^

Despite these favorable attitudes and the promise of restoration, speech‐decoding research has not met with universal enthusiasm. This is because the same research program is viewed by others as a fundamental threat to mental privacy.

In the seminal 1890 article “The Right to Privacy,” Samuel Warren and Louis Brandeis outlined a new and expansive conception of privacy that would become a cornerstone of modern privacy law in liberal‐democratic society. This right was articulated on the basis of “the right to be let alone” and “the right to one's personality”—the idea that we should not be compelled to disclose information about ourselves that we would prefer to remain concealed.^14^ In developing this work, Warren and Brandeis were motivated—at least in part—by the novel threats that accompanied emerging technologies of the day.^15^ Two years before the publication of “The Right to Privacy,” the first portable and affordable camera—the Kodak—was invented, and amateur photographers were, for the first time, able to move through public spaces while surreptitiously documenting the lives of others. As a novel type of information‐generating technology that produced data of a personal nature, the Kodak evoked privacy concerns in at least two dimensions. The first relates to data security (to use modern parlance)—that is, the ways in which personal information is treated once it has been generated and the question of who presides over this process. With the Kodak, the photographer—not us—gets to decide whether data about ourselves is created, how it gets broadcast, and to whom. In this way, photography assumes a coercive quality, infringing on our agency and autonomy. The second dimension relates to a deeper notion of mental privacy. It asks, what is the nature of the data being generated, and what information about us is inherently housed within it? As an innovative technology, the Kodak was capable of capturing our physical likeness and facial expressions with a degree of fidelity that eclipsed contemporaneous technologies, generating a novel form of personal data with the potential to reveal intimate and sensitive information. These two dimensions—a lack of agency over the creation and dissemination of data about us and, second, the detailed and personal nature of the data itself—compounded to render the Kodak a novel affront to people's private lives.

When speech BCIs are judged solely along the former dimension (relating to data security), the privacy threats they pose are largely familiar. In today's digital age, we are well accustomed to technologies as creators and purveyors of personal data, often handling ours in ways over which we have little to no control. Given the proliferation and commodification of personal data accrued online, speech BCIs could simply be viewed as an iteration of these technologies—the next data‐making devices contributing to a sea of existing personal information. However, when we consider the affront to privacy posed by speech BCIs along the latter dimension—that is, in light of the information housed within the neural data itself—the true extent of these concerns come to light. Just as the Kodak represented an informational step change in the ability to generate data detailing our physical likeness, speech BCIs may represent a step change in the ability to generate data that would reveal the fine‐grained details of people's inner mental lives.

Even if we restrict our comparisons to the domain of biosensing devices, speech BCIs are plainly a step change in the ability to reveal detailed contents of one's inner mental life. An array of devices on the market, from pacemakers to wearables, accrue signals that enable inferences about users’ physiological and psychological states. In the neural domain, BCIs have been developed since the 1970s to decode relatively rudimentary information that would allow, for instance, brain control over a cursor moving on a screen.^16^ Today, commercially available neural devices decode so‐called global brain states—slowly unfolding information that provides a gross indication of users’ levels of fatigue or focus. Of course, these devices enable powerful inference into users’ mental states and understandably elicit privacy concerns of their own.^17^ However, as an information‐bearing signal, speech is unique in its density. As a result, speech BCIs far outstrip prior biosensing devices in terms of sheer bandwidth—the rate at which bits of information are sensed and decoded. Additionally, speech decoding necessarily interfaces directly with the neurons that house linguistic information, and because language is intuitively regarded as proximate to thought, concerns about surveillance are a natural product of the belief that—whether intentionally or inadvertently—speech BCIs may interface with the physical substrate from which our private thoughts emanate.

## Broader Concerns about Speech Decoding

The surveillance concerns we've outlined above stem from what might be considered “intrinsic” features of speech decoding as a biotechnological phenomenon. These intrinsic features then intersect with extrinsic societal, historic, and economic factors in ways that amplify those fears. Here, we consider three such factors as illustrative: First is a recent strain of media coverage common to several emerging technologies that combines unbridled technical optimism with dystopian pessimism, envisioning radically negatively transformed futures. Second are negative historical precedents in which medical treatments and experimental technologies have in fact been developed in ways exploitative of or otherwise harmful to disadvantaged groups. Finally, there is a currently uncertain but rapidly evolving industrial‐economic landscape that surrounds speech‐BCI development.

We noted above how media portrayals of research centered around the interests of people with disabilities can bolster support for restoration. There are also structural features of the present media landscape that direct coverage toward narratives emphasizing concerns about surveillance. We now live in an era that has been politically and economically transformed by the near‐universal adoption of personal mobile computing and digital social media, with wide‐ranging and destabilizing effects that were essentially impossible to predict at their outset. Our recognition of these transformations in hindsight has created a powerful incentive for media producers and consumers to be early in identifying the next technocultural paradigm shift that will similarly alter our lives. Recent subjects for this hype dynamic that so far have not made the foretold transformation in most people's lives, despite billions in investment, include direct‐to‐consumer genomics, virtual and augmented reality, coworking spaces, blockchain, microfluidic diagnostics, and self‐driving cars. A curious feature of some of this media coverage is how impressions of the inevitability of technology‐fueled transformations can be stoked by anxieties that take for granted the technical efficacy and widespread general use of these technologies. For example, the availability of consumer genomic technologies so far has not widely led, as some earlier commentators suggested it would, to genomically based social stratification or genetic manipulations to produce “designer babies”—the genome has proven to be much more complex and ambiguous than originally envisioned, and even more mundane applications like drug discovery or personalized health care have so far been commercially unsuccessful. More recently, it has been observed that a “doomerist” strain of fears about AI is actively encouraged by some AI developers and appears paradoxically to serve their business interests, perhaps in part because dystopian fears about AI bolster a supposition that the underlying technology will be powerful and ubiquitous.^18^

Turning specifically toward media coverage of speech decoding, fears about surveillance are a species of ethical consideration that can play a similar role in presenting neurotechnology‐driven cultural change as inevitable. Consider a 2023 *Vox* news site article that opens with the headline “Mind‐Reading Technology Has Arrived: An AI‐Powered ‘Brain Decoder’ Can Now Read Your Thoughts with Surprising Accuracy.”^19^ In this presentation, scientific limitations and technical obstacles to progress are glossed over, such that speech decoding is not presented as a nascent research program but instead as an established fact. The potential scope of technology‐driven transformation is also widened: rather than focus on intended applications for the small fraction of the population with severe speech‐motor disorders, the headline makes the envisioned subject (or target) of technology any potential reader of the article. Thus, while narratives about restoration may promote emotional identification with individual users with disabilities and showcase the specific intended use of speech BCIs for assistive communication, here we see that a narrative about surveillance, at least within a frame of anticipating technologically driven cultural transformation, may envision indiscriminate application to the broadest possible segment of the population.

Beyond these broad media representations directed to the public at large, a second factor magnifying concerns about surveillance is the historical experience of specific communities previously targeted for surveillance and social control. Native Americans have long been targeted by the deployment of Western medicine and education to specifically undermine traditional customs and ways of life, leaving painful legacies of weakened attachments to families, native languages, and spiritual practices.^20^ Meanwhile, as Harriet Washington documents in *Medical Apartheid: The Dark History of Medical Experimentation on Black Americans from Colonial Times to the Present*, Black Americans have experienced a horrifying history of exploitation in medical research and of medical harms inflicted on them in support of a racist social structure.^21^ Concerns about neurotechnology may hold a particular historical resonance for Black Americans.^22^ Black prisoners and psychiatric patients were targeted in the promotion of ablative psychosurgery,^23^ and in the 1970s, a research program on applying psychosurgery and electrical brain stimulation to control urban unrest was funded by the National Institute of Mental Health and the Nixon administration's Department of Justice. The implications of a research program postulating Black neurologic dysfunction rather than White racism as a root cause of urban violence and promoting technological control over the brain as a means for minimizing civil disturbance were examined in an investigative report in the Black American magazine *Ebony* and related articles in Black newspapers.^24^ This research program was opposed by many civil rights organizations, including the NAACP, the Black Panther Party, the Mexican‐American Political Association, the American Civil Liberties Union, the National Organization for Women, and Students for a Democratic Society.^25^ The Congressional Black Caucus led opposition to psychosurgery at the federal level, though ultimately regulations on psychosurgery were generally passed in state legislatures following the examples of Oregon and California.^26^ Though these controversies are now distant, a shared memory of them and their connection to ongoing experiences of racism may make concerns about surveillance via neurotechnology especially salient for members of historically targeted groups.

A third extrinsic factor that amplifies concerns about surveillance is the currently uncertain but rapidly evolving industrial‐economic landscape surrounding speech‐BCI development. While there is clearly enthusiasm for neurotechnology among both the public and some investors, further advances will require enormous investments, given the many remaining engineering, neuroscientific, medical, and regulatory challenges that must be overcome before the technologies can be deployed outside highly specialized research contexts. Since relatively few people have severe speech‐motor disorders, it is unclear that the market for restoration is large enough to provide a business case that can justify such expenditures. This invites a skeptical reading in which support for restoration merely provides ethical cover for commercial forces whose true interests involve surveillance.^27^ After all, several of our most powerful digital technology companies owe their market position to their obsessive management of and control over vast quantities of personal information surreptitiously collected from users, and such companies might view neural data as another valuable resource to be incorporated into their processes for targeted advertising and behavioral prediction. The most prominent neurotechnology company developing BCIs is Neuralink, founded by Elon Musk, which has been notoriously opaque about its processes, scientific findings, and business goals. Neuralink's messaging to different audiences has been wildly inconsistent, in some cases prioritizing restoration for people with disabilities and at other times envisioning exotically transformative scenarios inspired by science fiction, including wide‐scale human enhancement and the merger of human intelligence with AI.

Together, then, a variety of factors inform ethical opposition to speech‐decoding research, including factors intrinsic to speech decoding as a biotechnological phenomenon as well as extrinsic factors reflecting the broader historical, media, and commercial context in which this work takes place. It is conceivable that what we have labeled “intrinsic” factors could be partially allayed by the research community; perhaps researchers could give credible assurances to the public that they will not develop technologies that enable surveillance as a potential application. However, the extrinsic considerations are largely independent of the trustworthiness and conduct of the speech‐BCI research community itself. For this reason, regardless of any assurances that the research community might give to the broader public, concerns about surveillance are likely to play a significant role in shaping future public perceptions of speech‐decoding research.

## Mapping Concerns to Neural Systems That Support Language

Thus far, we have described a contested space in which conflicting moral responses to research are driven not only by features of the research itself but also by the complex media, historical, and commercial contexts in which the research is evaluated. How can these differences be addressed productively?

A growing body of literature on *neurorights* offers one approach.^28^ These efforts largely operate downstream from the initial research and development of neural devices by creating robust data‐governance frameworks that would protect users’ privacy in the event that privacy‐infringing devices indeed exist. Such policies may, for instance, compel neurotechnology companies to develop user‐data agreements with opt‐out features or limit centralized neural data processing to mitigate the risk of breaches.^29^ As research continues to drive speech BCIs closer to market adoption, downstream approaches such as those offered by neurorights will become increasingly relevant for addressing concerns about surveillance while promoting restoration.

However, as a complementary approach that has received relatively little attention, we now turn upstream toward the science of speech decoding and to what in fact occurs at the interface between brain and device. In particular, we will focus on the neuroscientific factors relevant for discriminating the extent to which a putative speech BCI would decode communicative versus private information.

To achieve this, we must first outline what is known about the computational steps by which the neural circuits for speech and language transform an abstract thought into a precise speech act. This transformation occurs via a series of neural processing stages that are hierarchically organized, such that progressively later stages—those that are more proximate to the eventual production of an utterance and constitute “lower” levels of the hierarchy—receive input from those above. At the “highest” level of this processing hierarchy, a thought originates within the semantic network—a constellation of neurons broadly distributed across the cerebral cortex. Because activity within this network corresponds to the very earliest stages of neurolinguistic processing, information is yet to exist as “speech” and is instead abstractly represented as pure concepts and their relations.^30^ In contrast, at the lowest level of the hierarchy, neurons in a specific subregion of the motor cortex contain articulatory representations of speech. The activity of these neurons directs highly specific kinematic trajectories—motor commands that are passed to muscles in the vocal tract, enabling the speech articulators (the tongue, lips, jaw, and larynx) to move in a precisely coordinated fashion to produce a desired utterance.

To illustrate the difference between high‐level semantic and low‐level articulatory representations in the brain, consider that a bilingual English and Chinese speaker will have a concept of *woman* derived from their semantic knowledge of women and that this semantic concept will remain relatively stable across English‐ and Chinese‐speaking contexts. Also tied to the concept, however, are auditory, motoric, and phonological representations of *woman* that differ depending on whether the concept is being deployed in the context of speaking English or Chinese. In other words, across the two languages, we would expect to find invariant neural representations of *woman* at the level of semantic‐network activity but different neural representations at the level of articulatory activity within the motor cortex. In this way, at the highest level of the neurolinguistic hierarchy, semantic network activity represents the abstract conceptual origins of a thought, while at the lowest level, representations in the motor cortex are a highly specific articulatory realization of the forthcoming speech signal. Sandwiched in between are numerous intermediate representations at intervening stages along the thought‐to‐speech transformation, such as representations at the lexical and phonological levels. However, we will focus on the high and low extremes of the hierarchy (semantic and motor processing) to illustrate the ethical significance of spoken language's hierarchical organization in the brain.

In principle, a speech BCI could generate output by reading brain signals corresponding to any level of the linguistic hierarchy, yielding a multitude of plausible decoding strategies. In reality, however, scientific understanding of neural processing at higher hierarchical levels remains relatively poor, and the majority of research programs target low‐level representations—with the decoding of articulatory representations in the motor cortex yielding particularly promising results.^31^ Typically, under such a strategy, the intended kinematic trajectories of speech articulators are decoded first, before an additional mathematical model infers words and sentences from these trajectories.

Despite the success of this low‐level motor strategy and the relative lack of knowledge about high‐level representations, motor decoding of speech is not the sole focus of research. Indeed, research has demonstrated that meaningful speech can be constructed by decoding brain signals in the semantic network.^32^ Because these signals are situated far upstream from eventual speech outputs, the semantic strategy, unlike its motoric counterpart, does not produce decoded content that strictly adheres to users’ intended message in a verbatim fashion but, rather, captures the general meaning, or gist, of a thought.

The two decoding strategies contrasted here—motor versus semantic—may differ along many more dimensions, such as the type of neural measurement technology required or the quantity of neural data required to train learning algorithms in order to achieve a benchmark level of accuracy. For our purposes, however, the most ethically germane distinction between motor and semantic strategies is the extent to which they read brain signals that explicitly hold linguistic information with communicative intent. Due to this distinction, the decoding strategy employed by a given speech BCI affects the extent to which it might serve restoration alone or might additionally permit surveillance.

As previously mentioned, articulatory processing occurs at the lowest level of the linguistic hierarchy, constituting the final cortical processing stage before commands are relayed along nerve fibers that innervate muscles in the vocal tract. These articulatory brain signals are generally characteristic of willful and volitional attempts to vocalize. That is, in the absence of a deliberate speech attempt, most studies indicate, the brain does not robustly initiate an articulatory program. Motor neurons are only weakly activated, and the possibility of successfully decoding any message is greatly reduced.^33^ This may strike one as intuitive when considering the proximity between the motor neurons whose activity is being read and the vocal tract itself—as close as two synaptic connections.^34^

In contrast, the semantic strategy, by virtue of the fact that it interfaces with brain signals at a higher level of abstraction, is more prone to decoding information that a user might wish would remain private. While articulatory brain signals are characteristic of explicit speech attempts, semantic signals are active across a range of cognitive operations, including some unrelated to communicative tasks. Such signals can be described as “abstract” and “amodal,” in that, while they vary with the semantic content being represented, they remain relatively stable across the different modalities in which such content might be deployed. As a consequence of this abstraction, semantic decoders may generalize across cognitive tasks. For instance, a BCI system trained to decode users’ intended speech from the semantic network may also decode “speech” during altogether noncommunicative scenarios, such as while a user watches a movie, listens to someone else speak, or simply thinks privately.

A significant caveat to the claims above is that the semantic strategy is at a much earlier proof‐of‐concept stage of research and development than are motor strategies, the latter of which yield more accurate decoding and are currently being deployed in clinical trials with people with speech‐motor disorders. Nonetheless, early proof‐of‐concept studies demonstrate that semantic decoding is a very active research program with solid grounding to support further advances.^35^

As we have described, concerns about surveillance are primarily rooted in a belief that speech decoding will encroach on private thoughts. Our analysis of the functional distinctions among decoding strategies serves to more precisely locate these concerns. Where low‐level motor strategies are employed, surveillance concerns may be softened by the more explicitly communicative nature of these neural signals. (Of course, the security and governance of data generated by speech BCIs still raise other familiar ethical and privacy issues; these are generally not specific to speech‐decoding research and are shared with other research programs involving sensitive user data.) Meanwhile, semantic decoding is, by virtue of the signals decoded, more prone to developing in ways that may serve both surveillance and restoration. This is not because semantic decoding necessarily entails the decoding of private information, but merely because there are not analogous technical and neuroscientific grounds for reassurance that the decoded information is intended for communication in the way that articulatory signals are.

## A Research Program on Communicative Intent

A simple response to the presented dichotomy may already have occurred to some readers. If low‐level articulatory decoding strategies promise the benefits of restoration while skirting the dangers of surveillance, whereas high‐level semantic decoding strategies naturally raise greater concerns about surveillance, could we just restrict our support to the former low‐level strategies and withhold support from strategies at higher levels? This functional distinction is tenable, but unfortunately, it cannot fully address the ethical tension, and, in our view, its simplicity would come at significant cost for multiple reasons.

First, there are many people with speech‐motor disorders for whom approaches wholly reliant on articulatory decoding will be unsuccessful. Some neurologic disorders causing anarthria and speech apraxia—including cortical strokes of the speech‐motor cortex, some presentations of ALS, and variants of primary progressive aphasia—result in injury to neurons in the speech‐motor cortex. For many people with these conditions, articulatory brain signals will not be sufficiently robust to support reliable decoding, and speech restoration will likely require decoding strategies that leverage signals at higher levels of the neurolinguistic hierarchy.

Second, even for conditions for which articulatory strategies succeed, there are sound reasons for not presuming that restoration would be completely served without surveillance. It remains empirically unsettled to what extent articulatory decoding strategies exclusively decode intended speech or may also decode signals arising from noncommunicative “inner speech.” As previously described, the majority of research suggests that a physical attempt to speak is required for the activation of the speech‐motor cortex to levels sufficient for permitting articulatory decoding.^36^ However, a recent study complicates this picture, reporting that speech‐motor activity continues to be decodable during fully “imagined speech” or even during private “inner speech,”^37^ though these signals are fainter and modified in format in comparison to those elicited during deliberate speech attempts. Some discrepancies among these studies may eventually be explained by subtle variations across participants in the sampling of neuronal populations. However, given the current lack of consensus, considerations that rest wholly on neuroanatomic and linguistic‐hierarchical distinctions between decoding strategies are unlikely to enable a full dissociation between neural signals usable for surveillance versus restoration.

Given this, we do not support either a blanket promotion of articulatory decoding over other strategies or a blanket prohibition of semantic decoding. However, our examination calls attention to an ethically relevant feature of speech‐related neural signals that deserves consideration as a target of neuroscientific and neuroethical inquiry—the extent to which those signals being decoded are associated with communicative intent.^38^ We have already noted that the presence of robustly activated articulatory signals in the lower levels of the neurolinguistic hierarchy can help to indicate such intent. Meanwhile, the difficulty with higher‐level semantic signals is not that they are necessarily private but, rather, that they are not necessarily communicative.

For intermediate neural processing stages that occur between semantic and articulatory processing, it is as yet unclear to what extent the presence of detectable signals at these levels also reliably indicates communicative intent and whether there are other categorical differences between communicative and private signals, including the possibility that decoders trained on the former fail to generalize to the latter, providing a natural safeguard against encroaching on private thoughts.

We therefore regard communicative intent as an urgent topic for neuroscientific investigation in speech‐decoding research and also for conceptual and user‐experience investigations. Conceptually, more work is needed to refine what we admit is still a hazy notion. This notion, particularly as applied to speech processing arising within hierarchically organized levels, intersects with long‐standing and thorny philosophical problems of intention applying under some descriptions or at some levels of generality and not others.^39^ For instance, in many slips of the tongue, our representations at the articulatory level fail to conform to our intentions at the word or phonological level (as in phonemic paraphasic errors and speech praxis errors), or our representations at the word level fail to conform to our intentions at the semantic level (as in semantic paraphasic errors). Successful control systems for speech neuroprostheses may ultimately need to account for competing descriptions of speech intention and the distribution of errors across levels, in which case their designers will need to consider how such errors should be attributed and addressed.

In the domain of user experience, attention to communicative intent will require active engagement with prospective users about how speech prostheses will fit into their lives, considered holistically. Dimensions along which such user engagement is required include deciding on trade‐offs between fluency, accuracy, and verifiability of communicative intent, and how aggressively to make use of predictive autocomplete and autocorrect functions by way of large language models. Ultimately, the usefulness and usability of speech neuroprostheses is unlikely to be measurable purely in terms of technical metrics such as words per minute (though we know from prospective users that fluency is one highly valued consideration) but, instead, in terms of how such prostheses satisfy users’ needs for connection alongside communication and advance their broader human values. A core slogan of disability activism is “Nothing about us without us.” One measure of the sincerity of researchers’ commitment to restoration focused on users’ needs will be the extent to which these researchers actively solicit such input and incorporate it into the design of proposed interventions, to avoid the pitfall of designing devices about people with speech disabilities without directly engaging their concerns and priorities.

Finally, at the broader level of public discussion and debate, we hope that attention to communicative intent by investigators themselves, as well as by university press offices, journalists, media outlets, and commentators, can improve discourse about speech decoding. We recognize that the complexity of language neuroscience and variation across different research programs present serious challenges for public scientific communication about a topic of growing popular interest.

For journalists and others engaged in translating neuroscientific advances for broader understanding, we propose three basic questions for evaluating new speech‐decoding research that can help to situate findings in relation to the ethically conflicting applications of restoration and surveillance. First, from which regions of the brain were signals being decoded? As discussed, for signals arising in low‐level regions that are synaptically proximate to the vocal tract, surveillance would be less likely given the characteristically communicative nature of these neural signals. Such regions are variously referred to in neuroscience as the “ventral sensorimotor,” “speech‐motor,” or simply “motor” cortex.

Second, did investigators evaluate whether decoding models that were trained to decode explicitly communicative signals generalize to signals without such intent? Or do they provide other assurances that decoded outputs were indeed intended for communication? These questions are particularly important for signals decoded from higher‐level semantic networks—which can include a broad spatial extent of the cerebral cortex encompassing portions of the frontal, temporal, and parietal lobes.

Third, as studies move beyond proof of concept to applications, to what extent have the decoding system and metrics for its assessment been designed with the active input of an intended user population of people with speech disabilities? Research purely guided by technical measures of speed or investigator‐defined accuracy may not be guided by users’ own priorities in restoration, particularly when trade‐offs would be necessary among different decoding parameters in order to serve users’ needs. Focusing on such details may help science communicators to determine when narratives focused on restoration or on surveillance are more apt for guiding public understanding of the implications of a new research finding.

Though attention to the communicative intent of decoded information will not dissolve the ethical tension between support for restoration and concerns about surveillance, we believe that it can help to clarify the competing moral concerns at stake in responses to speech‐decoding research. Beyond this, we hope to have illustrated how neuroscientific details, such as regarding the hierarchical organization of the neurolinguistic system, can inform our interpretation of what might otherwise appear to be purely abstract neuroethical arguments regarding disability or mental privacy. Further work to examine and refine the notion of communicative intent, applying neuroscientific methods of discovery alongside conceptual inquiry and user engagement, may prove essential to navigating the challenges raised by this emerging area of neuroscience research.

## Acknowledgments and Disclaimer

This article is part of a series, Neuroscience and Society, whose development by The Hastings Center for Bioethics is funded by the Dana Foundation.

Support for the authors was provided by the University of California, Berkeley, Kavli Center for Ethics, Science, and the Public with a generous gift from the Kavli Foundation, by the National Institute on Aging of the National Institutes of Health under award K24AG083117, and by the National Institute of Mental Health of the NIH under award R01MH137095. The content is solely the responsibility of the authors and does not necessarily represent the official views of the Kavli Foundation or the NIH.
